# What role did serious mental illness play in Jackson Pollock’s drip paintings? Abstract expressionism and possible links to serious mental illness and encrypted images (polloglyphs)

**DOI:** 10.1017/S1092852924002347

**Published:** 2025-01-13

**Authors:** Stephen M. Stahl, Debbi Ann Morrissette, Jahon Jabali, Jon A. Gates

**Affiliations:** 1 Department of Psychiatry and Neuroscience, University of California Riverside, Riverside, CA, USA; 2 Department of Psychiatry, University of California San Diego, San Diego, CA, USA; 3 Neuroscience Education Institute, Malvern, PA, USA; 4 Arbor Scientia, Carlsbad, CA, USA

**Keywords:** psychosis, creativity, pollock, drip paintings, abstract expressionism, polloglyphs

## Abstract

The link between creativity and serious mental illness (SMI) is widely discussed. Jackson Pollock is one example of a giant in the field of art who was both highly creative and experiencing an SMI. Pollock created a new genre of art known as abstract expressionism (“action painting”) defined as showing the frenetic actions of painting. The question arises whether his SMI played any role in the way he created his drip paintings, especially when he was overactive and manic. Furthermore, did visual hallucinations or enhanced visual perception associated with mania or psychosis facilitate Pollock in embedding and camouflaging images under layers of thrown paint? Seeing images in Pollocks drip paintings has been a controversy ever since these paintings were created. Some experts attribute this to pareidolia—perceiving specific images out of random or ambiguous visual patterns—a phenomenon known to be enhanced by fractal fuzzy edges such as seen in Rorschach ink blots as well as in Pollock drip paintings. So, are Pollock’s drip paintings merely giant Rorschach images, or did Pollock insert polloglyphs—images that are encrypted that tell a story about Pollock’s inner being—into his paintings and then disguise them with drippings? Here, we explore answers to these questions and discuss images that Pollock included in his earliest sketches and used repeatedly in his abstract paintings and later in his drip paintings to argue that these images are not accidental.

## Introduction

Chronic and severe mental illnesses such as bipolar disorder and schizophrenia often have poor outcomes, especially if available treatments are not implemented and housing is not consistent.^1^ ^,^^2^ On the other hand, numerous observers have long noted the shared genetic vulnerability of serious mental illness (SMI) disorder with creativity,^3^ ^–^^13^ with this observation attributed to Aristotle himself: “no great genius has ever existed without a strain of madness.” The many artists, writers, and celebrities are said to have an SMI including Vincent Van Gogh, Edvard Munch, and Jackson Pollock.^11^ ^,^^12^ Here, we explore the relationship of Jackson Pollock’s disorder both to the manic energy he exhibited while painting his drips and the proposal that he used symbolic images across his lifetime to articulate his unconscious thoughts and conflicts within his art.

Experts have been fascinated with Jackson Pollock (born 1912, died 1956) and the meaning of his famous “drip paintings” ever since he began producing them in the 1940s.^14^ ^–^^19^ Winston Churchill used the phrase “a riddle, wrapped in a mystery, inside an enigma” to describe a situation that was difficult to understand. This quote can undoubtedly be applied to the 70+ years of attempting to comprehend the meaning of Jackson Pollock’s famous drip paintings. Some of the currently unresolved questions include: Is there more to them than just the representation of the motion and frenetic activity that occurred during the act of painting, often given as the classical explanation of his genre, abstract expressionism^14^ ^–^^19^? Do his drip paintings incorporate images he expressed in earlier work and possibly arising from his troubled and chaotic inner world? If so, are these images accidental, unconscious, or purposeful to tell a story?

### Did Jackson Pollock have an SMI and was it linked to his creation of drip paintings?

**Figure 1.**
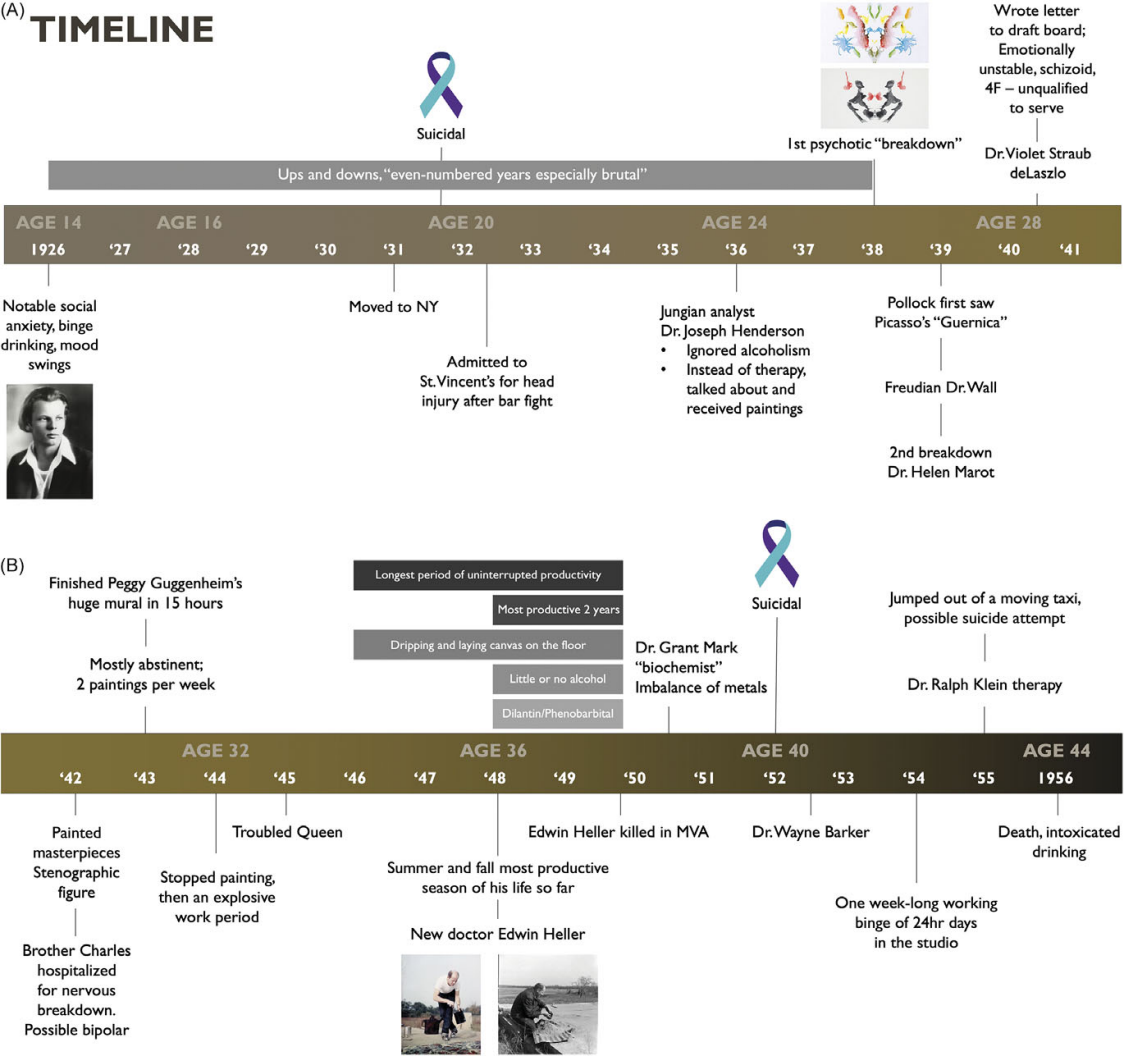
(A) Timeline of psychiatric events for Jackson Pollock, ages 14 to 28 (1926–1941). (B) Timeline of psychiatric events for Jackson Pollock, ages 30–44 (1942–1956).

It is well documented that Pollock began to have mood swings as a child, with symptoms of social anxiety relieved by alcoholic binges from his teen years until his death (see timeline in Figure 1A).^14^ ^–^^19^ He received psychiatric treatment from age 23 until his death at age 44 (Figure 1B), mostly in the form of outpatient psychoanalytic psychotherapy, with several psychiatric hospitalizations and some psychopharmacologic treatments (Figure 1A and Figure 1B).^19^ Most of his treatment was ineffective by psychiatrists trained in Jungian or Freudian psychoanalysis and at times homeopathic practitioners prescribing “healing minerals”.^19^

Box 1 or Table 1: Glossary.

**Abstract expressionism**—the representation of free, spontaneous, and personal emotional expression that occurred during the act of painting.

**Fractal edges**—uneven edges with random patterns which can provoke viewers to see images. Rorschach inkblots have fractal edges and are used in psychiatry to provoke the patient to project images onto the inkblots and thereby determine what the patient is thinking to help make a diagnosis.

**Pareidolia**—perceiving specific images out of random or ambiguous visual patterns.

**Polloglyphs**—images arising from Jackson Pollock’s conscious or unconscious creativity, and embedded in his paintings, especially his drip paintings, often camouflaged with layers of dripped paint and often to tell a story about something significant in his life.

**Figure 2.**
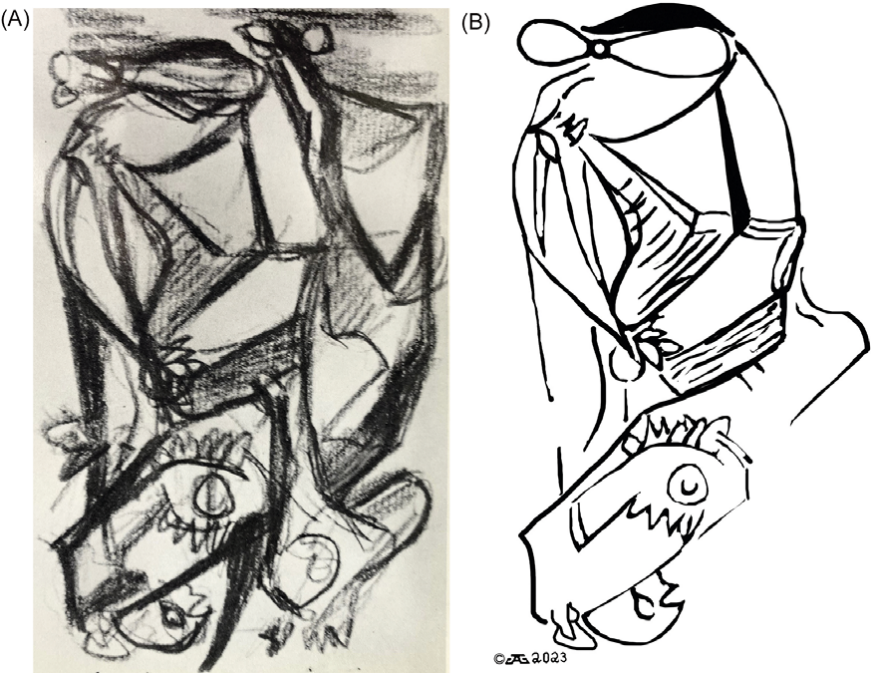
shows “drunken monkey” untitled sketch from the Wysuph sketchbook utilized by psychiatrist and psychoanalyst Dr. Joseph Henderson to interpret Jungian psychoanalytic conflicts. Plate 76 in reference 18. (A) Drunken Monkey rotated 180 degrees (upside down). Here, a monkey with glasses can be seen holding a wine bottle with another booze bottle visible. (B) Drunken Monkey with outlines of the embedded images of the monkey, booze and wine bottles, without the background of the original sketch. Compare Figure 2A and 2B to better visualize the embedded images.

Pollock’s first psychoanalyst was Joseph Henderson, who began treating Pollock during his first year in practice and diagnosed Pollock with schizophrenia and alcoholism (Figure 1A).^19^ Henderson found that Pollock was not very verbal about his problems or drinking habits so they communicated by means of artistic sketches. The 83 sketches on 69 pieces of paper published in Wysuph’s book^18^ were thought by Henderson to represent Jungian symbolism and illustrate Pollock’s psychiatric illness and unconscious conflicts.^18^ ^,^^19^ Henderson also took possession of Pollock’s sketches as a form of payment while ignoring doctor–patient confidentiality. However, Henderson wrote and lectured extensively on his sessions with Pollock. He also sold these “therapeutic” drawings for his fame and profit once Pollock became prominent.^14^ ^,^^18^ ^,^^19^ Likely, these “sessions” with Henderson were not therapeutic for Pollock, as discussing art in a Jungian psychoanalytic setting today would be considered ineffective treatment if not unethical practice for his disorder. In fact, while working with Henderson, Pollock deteriorated. He was psychiatrically hospitalized for the first time at New York Westchester Hospital in 1938 at the age of 26 with his first “breakdown,” likely a manic or psychotic episode combined with alcohol intoxication.^19^ Without modern mood-stabilizing antipsychotic medications or lithium at the time, Pollock was treated mostly with rest.

Although according to his biographers, Pollock was variably diagnosed by his psychiatrists as “alcoholic psychosis,” “schizoid,” or “a schizophrenia like disorder characterized by alternating periods of violent agitation and paralysis or withdrawal”.^19^ In today’s world, he would more likely be diagnosed as bipolar. That is, Pollock did not experience the classical paranoid delusions and auditory hallucinations with thought disorder that are now the core diagnostic criteria for schizophrenia.^2^ Instead, he experienced very unstable moods with devastating periods of depression alternating with high energy, irritable mania, and hypomania while he self-medicated with huge intakes of alcohol.^19^ For example, when Jackson Pollock was living with his brother Sanford and Sanford’s wife Arloie, she recalls in Wysuph’s book^18^ a pattern of mood cycling consistent with bipolar disorder: “Pollock would become unusually quiet and depressed for a few days before going on a “binge.” The binge would in turn be followed by a period of solitude and depression. Then, sober, Pollock would begin a period of intense painting and drawing.” Pollock’s own wife Lee Krasner similarly noted that this very pattern “continued into the late 40s and that during periods of depression, Jackson would become so withdrawn as to be impenetrable”.^18^

This cycling of mood that Pollock experienced is typical of bipolar disorder in contrast to periodic episodes of paranoid psychosis with auditory hallucinations which is typical of schizophrenia and which Pollock did not experience. The diagnosis of bipolar disorder is further supported by other comments from Pollock’s biography^19^ that “more and more the schizophrenic like state described by his psychiatrist was playing itself out in a binary drama of depression and elation.” The observation that Jackson’s older brother Charles—who exhibited no signs of schizophrenia—was hospitalized in 1942 for a “nervous breakdown” possibly a bipolar episode is also suggestive of a positive family history of bipolar disorder in the Pollock family.^19^

During Pollock’s first psychiatric hospitalization, he was tested extensively with Rorschach ink blots, a new way of evaluating psychiatric disorders at the time and adapted from the 1921 publication of inkblots by the Swiss psychiatrist and psychoanalyst Hermann Rorschach.^19^ Exposure to Rorschach ink blots would have introduced Pollock to the phenomenon of pareidolia—perceiving specific images out of random or ambiguous visual patterns—which could have very well influenced Pollock’s drip technique of camouflaging Polloglyphs in his later works. It is now well-known that fractal edges—uneven edges with random patterns—can provoke viewers to see images.^20^ ^–^^23^ Rorschach inkblots have fractal edges and are used in psychiatry to provoke the patient to project images onto the inkblots and thereby determine what the patient is thinking to help make a diagnosis.

Pollock’s drip paintings also have fractal edges, and the question is whether fractal edges in his drip paintings are an accident of throwing paint, with unintended provoking of images in the observer, or if Pollock recognized that fuzzy edges would provoke images, so he did this on purpose as a component of a technique to consciously or unconsciously create camouflaged images. Art critics generally do not believe Pollock did this on purpose and tend to focus on Pollock’s actions during the performance of painting rather than on the images resulting from these actions.^14^ ^,^^19^ ^,^^24^ ^,^^25^ If the principal meanings of Pollock’s drip paintings are pictorial energy, and a new sense of motion, with the dripped line imparting a sense of constantly changing velocity, then Pollock’s bipolar disorder may have been at the heart of how he created this revolutionary new genre in the art world. That is, bipolar mania or hypomania, associated with increased activity, energy or agitation, likely was linked to the creation of Pollock’s drip paintings, perhaps nowhere better demonstrated than in Namuth’s famous film of Pollock painting where “it is almost impossible to keep track of where Pollock’s rapidly launched strokes are landing. The almost inevitable effect is to reinforce the cliché that he is flinging paint at random”.^14^ ^,^^19^ ^,^^24^ ^,^^25^

Pollock’s disorder could have also been linked to his embedding images and camouflaging them in his drip paintings. It is well documented that Pollock was afflicted with hallucinatory spells, particularly visual.^19^ With his eyes wide open, he would suddenly begin to see whirling images, and Pollock himself realized that for his drip paintings, he had seen those images before he painted them. Bipolar experts have written about altered sensory phenomena experienced in bipolar disorder and even theorized a suprasensory world for some patients with enhanced visual perceptual abilities especially when manic or hypomanic.^26^ ^–^^30^ Given that Pollock included images in his pre-drip paintings, and some of these same images occur repeatedly in several of his drip paintings, it is possible that Pollock’s bipolar visual perceptions allowed him to develop a unique technique to camouflage images beneath drippings. Pollock himself gave conflicting information on whether his paintings contained images, on the one hand stating, not only “I choose to veil the image”^24^ ^,^^25^ but also “I deny the accident” and “it took a long time to learn how to pour and drip paint like using a giant fountain pen” as though he was painting purposely in the air above the canvas.^19^

About 1947, Jackson Pollock began his drip paintings and his longest period of uninterrupted productivity until about 1950 (Figure 1B).^19^ During this period, he created his masterpieces, especially during the years between 1948 and 1950, a time when he drank little and was treated with the early mood stabilizers dilantin/phenytoin and phenobarbital.^14^ ^,^^19^ These agents are not effective for the treatment of schizophrenia but can improve bipolar disorder, particularly mania.^1^ ^,^^31^ Additional evidence is that Pollock had bipolar disorder and not schizophrenia. These observations of improved artistic output and productivity while undergoing effective bipolar disorder treatment support the proposal that serious and disabling mental illness such as bipolar disorder can nevertheless be associated with productive output from creative genius when properly treated. But for the treatment Pollock received during this time, Pollock’s greatest masterpieces of abstract expressionism in drip paintings might have never been created. Unfortunately for Pollock and his subsequent productivity, the only psychiatrist who prescribed effective psychiatric treatment for Pollock’s bipolar disorder was Edwin Heller, who unfortunately died after just two years of treating Pollock with mood stabilizers.^19^ After Heller’s death, Pollock stopped his medications, resumed drinking with the return of unstable moods and suicidal ideation, and lost his high productive output for the rest of his life despite seeing others for ineffective treatments and quackery.^19^ Pollock eventually crashed his car a few years later after drinking in a possible suicidal act and died at age 44.

### Pollock’s images in his sketches and paintings prior to his drip paintings

Although Jackson Pollock is most famous for his drip drawings, these occurred late in his career, starting around 1947. Prior to that he produced sketches for his psychoanalyst Henderson in the 1930s as mentioned above, and after that, several nondrip paintings including some “surrealist inflected” paintings and “gestural abstraction” paintings.^19^ ^,^^24^ ^,^^25^

**Figure 3.**
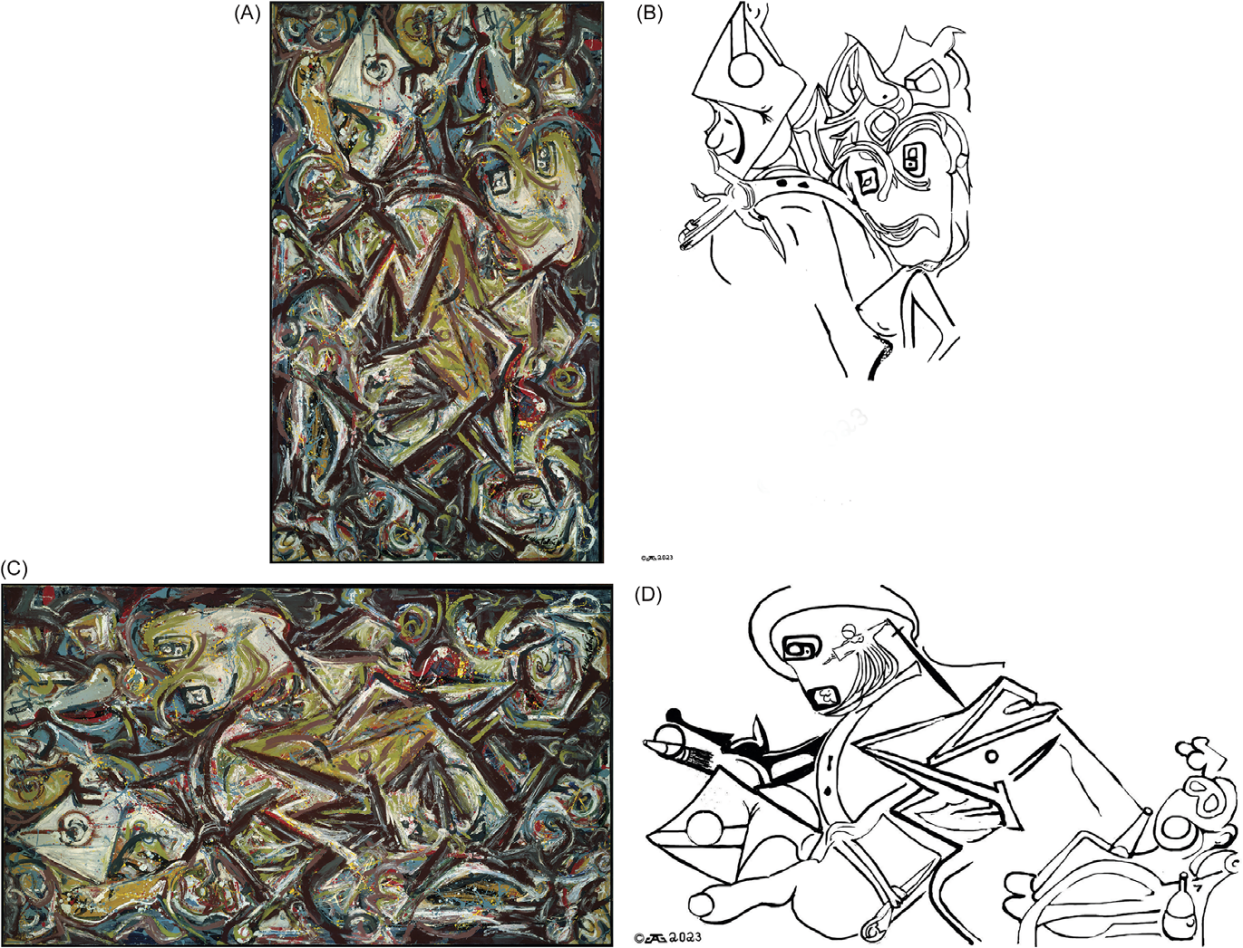
Troubled Queen (1945, Museum of Fine Arts, Boston). (A) Troubled Queen upright, original display. Not only a “troubled queen” but also another figure, possibly a soldier, and other images are shown. (B) Troubled Queen. Images of the troubled queen and a soldier in outline without the background of the original painting are shown. Compare Figure 3A and 3B to better visualize the embedded images. (C) Troubled Queen, rotated 90 degrees counterclockwise. A charging soldier holding a hatchet and a pistol with a bullet in the barrel; a Picasso-esque rooster; a monkey with goggles and wine; and one of the clearest images, the angel of mercy and her sword are shown. (D) Images outlined here without the background of the original painting show soldier, rooster, monkey, and angel of mercy with a sword. Compare Figure 3C and 3D to better visualize the embedded images.

**Figure 4.**
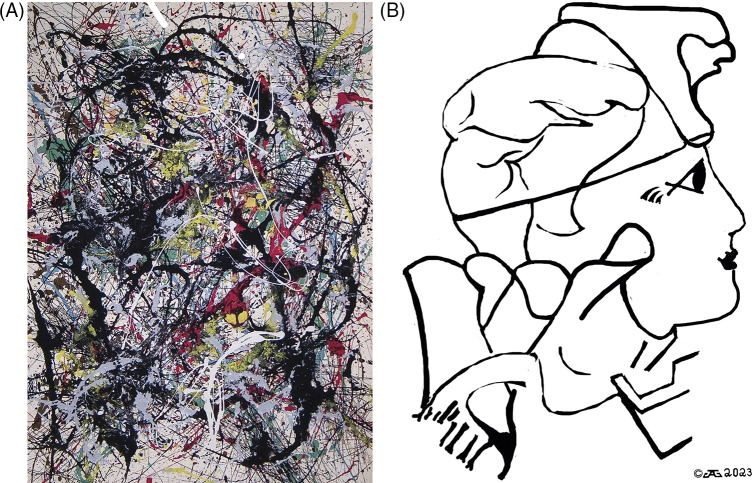
“Pretty lady” rotated 90 degrees clockwise (number 34, 1949 Munson-Williams-Proctor Institute Utica NY), one of his drip paintings, which we will call “Pretty Lady.” Pollock was rumored to be dedicated to the actress Lauren Bacall and one can see her image in this rotated drip painting (compare Figure 4A). Here is a profile of a pretty lady looking to the right. (B) The pretty lady is outlined here without the background of the original painting. Compare Figure 4A and 4B to better visualize the embedded images.

**Figure 5.**
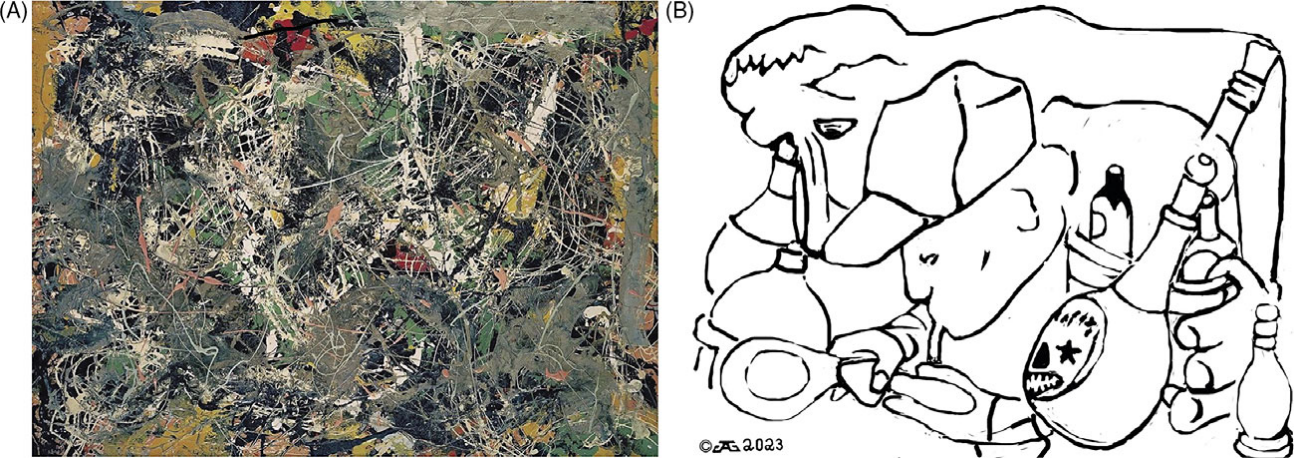
(untitled 1949, Foundation Beyeler Reihen/Belel Beyeler Collection, rotated 90 degrees clockwise) which we will call “Monkey on My Back” shows a smoking Pollock with his signature baseball cap looking at something with a magnifying glass; a monkey (or guerilla) with his arm around Pollock looking on; and also those notorious booze bottles. (A) shows the original rotated 90 where these images are visible (a smoking Pollock with his signature baseball cap looking at something with a magnifying glass; a monkey (or guerilla) with his arm around Pollock looking on; and also those notorious booze bottles. (B) These images listed in the legend for Figure 5A are outlined here without the background of the original painting. Compare Figures 5A and 5B to better visualize the embedded images.

One example of the earliest sketches of Pollock from the Wysuph book^18^ is shown here in Figure 2A which we will call “Drunken Ape” (18, plate 76 rotated 180 degrees). This sketch is best observed upside down from the way it is presented in the Wysuph book, a trick Pollock used throughout his career perhaps to better camouflage his images. Figure 2A shows a monkey along with a wine bottle and a booze bottle. An outline of these images is shown in Figure 2B and can facilitate recognition of these same images in the original sketch (compared to Figure 2A). Monkeys, gorillas, wine bottles, and booze bottles are seen not only in these earliest primitive sketches given to his psychoanalyst in the late 1930s but also throughout Pollock’s later abstract paintings and drip paintings (see Figures 2–5). Pollock was obsessed with drinking and was a very severe alcoholic,^19^ which could explain the recurrence of alcohol representations in his work. But what does the monkey represent? Pollock himself? Other sketches from the Wysuph book (18, not illustrated here) include “Highway Robbery,” plate 39 showing a shootout of banditos with the driver of a car with a little boy inside. Another example is “Car Crushed” (18; plate 15) showing what at first glance may look like a Native American sketch, but on closer analysis shows a car running over someone, perhaps Pollock, with paint brushes impaling the tire of the car, possibly to show that painting was literally killing him. Many other sketches contain additional images not only in the Wysuph book but also in subsequent paintings.

For example, not shown here is what we will call “Smoking Monkey” (actually untitled from 1938–41, Art Institute of Chicago) shows an upside-down monkey in glasses smoking a cigarette. Another example illustrated here in Figure 3 is the far more revealing set of images clearly present in the work titled “Troubled Queen” from 1945 and the Museum of Fine Arts Boston (Figure 3 ABCD). Troubled Queen is considered to be Pollock’s masterful transitional work from the regionalist figurative paintings of his early years to the passionate “drip paintings” for which he is best known.^19^ ^,^^24^ ^,^^25^ As stated by Elliot Bostwick Davis et al (mfashop.com/9020398034), “As Troubled Queen shows, Pollock had begun to work in a very large scale by this time; his paint was dragged over, dripped on, and flung at the canvas. His subject matter was no less highly wrought: emerging from the churning coils and jagged lines of this life-sized canvas are two facelike forms, one a leering mask and the other a one-eyed diamond shape. Their nightmarish presences reflect not only Pollock’s agitated psyche but also the years of violence that had torn the world apart through war” (see Figures 3A and 3B). Thus, Troubled Queen shows convincingly that Pollock included images, which we call Polloglyphs, in his painting prior to his “drip paintings,” rendering it feasible that he continued to include images in his later classical “drip paintings” using the new technique begun at this time.

Exploiting Pollock’s trick to better visualize and uncover many other polloglyphs, Troubled Queen can be rotated 90 degrees counterclockwise (Figure 3C) to reveal more images (Figure 3D) including a charging soldier holding a hatchet and a pistol with a bullet in the barrel; a Picasso-esque rooster; a monkey with goggles and wine; and one of the clearest images, the angel of mercy and her sword. Troubled Queen was completed in 1945, during World War II, and specifically after 1939, the time when Pollock first saw Picasso’s famous Guernica about the Spanish Civil War, which was painted in 1937. Pollock had been excused as unfit to serve in the US army during World War II due to his psychiatric disorder, and one wonders whether the images and timing of Troubled Queen could be telling a story of how Pollock felt about the war and might be his own “Guernica.” Pollock was obsessed with Picasso and felt an intense competition with him, always hoping to better that giant with his own work.

Although some of the later polloglyphs shown in later drip paintings and discussed below may be more difficult to see or “decode” from the chaotic layers of thrown paint serving as camouflage (see Figures 4 and 5), we propose that the images up to this point in Pollock’s career are not Rorschach ink blots with fractal edges fooling the eyes and only in the mind of the viewer but represent images purposely put on canvas. Clearly, there is a “troubled queen” in Troubled Queen.

Did Pollock suddenly stop including images in his work and move into abstract expressionism only representing frenetic action painting with no content and the drips only showing the aftermath of movement with fractal edges and false images? Or did he consciously or unconsciously embed Polloglyphs as well during his action painting to tell a story?

Art critics state “Once in a while a lifelike image appears in the painting by mistake. But Pollock cheerfully rubs it out because the picture must retain a life of its own”.^19^ ^,^^24^ ^,^^25^ Also, “the conscious part of his mind,” he says plays no part in the creation of his work. It is relegated to the duties of a watchdog; when the unconscious sinfully produces a representational image, the conscience cries alarm and Pollock wrenches himself back to reality and obliterates the offending form”.^24^ ^,^^25^ This is why experts not only do not believe there are any images in Pollock’s drips, and that any accidental fractal fake images (such as we identify in Figures 4 and 5) were neither conscious nor unconscious.

### Images in Jackson Pollock’s drip paintings—polloglyphs. Do these arise purposely from his conscious mind or from his repressed unconscious mind, or are they all in the viewer’s mind?

Figure 4 shows a 90-degree clockwise rotation of Pollock’s painting he numbered #34 (1949 Munson-Williams-Proctor Institute Utica NY), one of his drip paintings, which we will call “Pretty Lady.” Pollock was rumored to be dedicated to the actress Lauren Bacall and one can see her image in this rotated drip painting (compare Figures 4A and 4B).

Figure 5 (untitled 1949, Foundation Beyeler Reihen/Belel Beyeler Collection, rotated 90 degrees clockwise) which we will call “Monkey on My Back” shows a smoking Pollock with his signature baseball cap looking at something with a magnifying glass, a monkey (or guerilla) with his arm around Pollock looking on, and also those notorious booze bottles (compare Figures 5A and 5B).

These paintings and several more were presented at a psychiatric congress in November 2023,^26^ ^,^^32^ ^,^^33^ and the results of a survey of 48 mental health observers showed that a large majority75% felt that Pollock had bipolar disorder, whereas 85% saw images in Pollocks paintings with 58% seeing them in both Troubled Queen and in the drip paintings, and 27% seeing them in Troubled Queen but not in the drip paintings. Finally, 67% thought that images were purposely included by Pollock but disguised, whereas 17% thought the images arose from Pollock’s unconscious and even he was not aware of them and another 15% thought that the images were in the viewer’s mind not in Pollock’s paintings. Only 2% thought there were no images present.^26^ ^,^^32^ ^,^^33^

## Discussion

Hamlet: Do you see yonder cloud that’s almost in the shape of a camel?

Polonius: By th’ mass, and ‘tis like a camel indeed.

Hamlet: Methinks it is like a weasel.

Polonius: It is backed like a weasel.

Hamlet: Or like a whale?

Polonius: Very like a whale.

This famous dialog from Shakespeare’s Hamlet is an example of pareidolia and is used by Hamlet in this case to feign madness on his girlfriend’s father Polonius. Perhaps Shakespeare is also using Hamlet to play with the act of perception and ask how can a person ever know what is real? In this case, Polonius cannot be trusted; He is a sycophant, and Hamlet will stab him to death two scenes later.

Are polloglyphs present in Jackson Pollock’s drip paintings or are they the mad product of viewers’ misperceptions? Seeing images in Pollocks drip paintings has been a controversy ever since these paintings were created. So, was Jackson Pollock “Jack the dripper” with paintings “that a dog or cat could have done better,” or did Pollock insert polloglyphs—images that are encrypted that tell a story about Pollock’s inner being—into his paintings and then disguise them with drippings? On the one hand, some—especially art critics—have emphasized the formal elements of Pollock’s work, arguing that no images are present and viewers can find whatever they are looking for because such images are artefacts of the “fractal” fuzzy edges to the drippings and are just fooling the eyes. Thus, maybe Pollock’s paintings are just a massive set of new Rorschach inkblots to provoke the viewer to project their own emotions onto the painting, where there is actually nothing at all in the painting from the artist.

On the other hand, seeing an image once in a drip painting could be random; seeing the same image twice in different paintings could be a coincidence; seeing it three or more times, as is the case for booze bottles, monkeys/gorillas, elephants, and more, make those images very unlikely to be random provoked pareidolia. Furthermore, from a psychiatric point of view, given that Pollock had bipolar disorder, painted when he was euthymic or manic and not intoxicated nor depressed, had extensive exposure to Rorschach ink blots during his own psychiatric treatment, had visual images and hallucinations of images, clearly incorporated images into his predrip paintings (eg, see Troubled Queen Figure 3), and used repeatedly the same images in multiple drip paintings (eg, booze bottles, images of himself, monkeys, clowns, elephants, and more). The alternate point of view is that Pollock either consciously or unconsciously encrypted images in his drip paintings to tell us a story. His remarkable ability to do this with polloglyphs hiding in plain sight may be part of Pollock’s creative genius and could have been enhanced by the endowment of extraordinary visual–spatial skills that have been described in some bipolar patients.^28^ ^–^^31^ If so, painting could have been Pollock’s way to rapidly unspool his images and to do this onto canvas and thus communicate his personal stories.

In favor of the images being accidental, Pollock himself stated that consciously “I try to stay away from any recognizable image; if it creeps in, I try to do away with it”.^19^ However, he also admitted “recognizable images are always there in the end”.^19^ If coming from his deep unconscious creativity and genius, such images may have appeared in spite of himself. Pollock thus may indeed not have been mindful of creating Polloglyphs as he stated “When I am in my painting, I’m not aware of what I am doing”.^19^ He painted in air, letting gravity make the picture, and dripping became not just another way of obscuring images but also a new way of creating them. Ultimately, we may never know if there are polloglyphs present in Jackson Pollock’s famous drip paintings, nor can we know for sure whether they are merely in the mind of the beholder or put there consciously or unconsciously by the artist. In the meantime, it can be enlightening to view Pollock’s works and decide for oneself while keeping in mind that chronic and severe mental illnesses such as bipolar disorder can be associated with creativity and genius, with a good outcome, especially with effective treatment.

## Data Availability

The data that support the findings of this study are available from the corresponding author, Stephen Stahl, upon reasonable request.
